# End‐Of‐Life Simulation in Undergraduate Nursing Curricula: A Cross‐Sectional Survey

**DOI:** 10.1111/jocn.17776

**Published:** 2025-04-11

**Authors:** Cindy Hoang, Beverley Copnell, Sharon Bourke, Monica Peddle

**Affiliations:** ^1^ School of Nursing and Midwifery La Trobe University Melbourne Australia; ^2^ School of Nursing and Midwifery Deakin University, Centre for Quality and Patient Safety in the Institute of Health Transformation Burwood Australia

## Abstract

**Aim:**

The aim of this study was to explore end‐of‐life simulation in undergraduate nursing curricula in Australian and New Zealand institutions.

**Design:**

A cross‐sectional descriptive research design was employed. The study is reported using the CROSS checklist.

**Methods:**

A survey was distributed to 45 institutions with an accredited Bachelor of Nursing programme in Australia or New Zealand. The instrument comprised eight domains: simulation orientation, simulator type, simulation environment, instructional design, simulation event, pre‐brief, debrief, and facilitation preparation and requirements.

**Results:**

Thirty institutions responded to the survey, with 25 suitable for data analysis. Eleven institutions included end‐of‐life simulation in their curriculum. The dominant modality used in the end‐of‐life simulation was high‐technology manikins. All institutions used a validated approach to conducting the pre‐brief and debrief. Variations were reported in the skill and clinical expertise required of end‐of‐life simulation facilitators and the approaches and modalities used in end‐of‐life simulations across institutions.

**Conclusion:**

A small number of institutions reported including end‐of‐life simulations in their undergraduate nursing curriculum. This study found the end‐of‐life simulations integrated into undergraduate nursing curricula in Australia and New Zealand align with many elements of the Healthcare Simulation Standards of Best Practice. There were variations in the simulation modality and facilitation style used to deliver end‐of‐life simulations across institutions. While a pre‐brief session was included, the elements covered and information conveyed to participants varied across institutions. Additionally, the content expertise required of simulation facilitators lacked clarity.

**Recommendation for Future Research:**

The influence the pre‐brief has on the student learning experience requires further research. Moreover, the learning experiences of the participants in various simulation modalities, including the influence of SPs and debriefing approaches, warrant investigation. The role and impact of professional development and facilitator requirements, such as skills and clinical expertise, on the student learning experiences and outcomes in EOL simulation offer opportunities for further research.

**Patient or Public Contribution:**

There were no patient or public contributions in this study.

**Summary**

- Detailed information on the integration and implementation of end‐of‐life simulation in undergraduate nursing courses in Australia and New Zealand.
- The end‐of‐life simulations integrated in undergraduate nursing courses align with many of the elements in the Healthcare Simulation Standards of Best Practice.
- The clinical and educational requirements of end‐of‐life simulation facilitators are not consistent across educational institutions, which may impact the learning experience and preparation for the practice of students.

## 1 Introduction

End‐of‐life (EOL) care is an important aspect of palliative care. It refers to care provided to a person in their final stages of life and involves physical, psychological and spiritual aspects of care (Kim and Kim 2021). In acute healthcare settings, many of the interactions and conversations occurring at EOL are initiated by health professionals. Nursing staff in particular play a key role at EOL as they are often the health professionals providing daily assistance to patients and families (Hoang et al. 2022). In most developed countries, nurses are the largest regulated group of healthcare professionals to provide EOL care (Fristedt et al. 2021; Pereira et al. 2021).

Many health professionals do not have specialised training in EOL care (Witkamp Frederika et al. 2015), and thus may be underprepared to provide care at EOL. Palliative Care Australia (2022) argues that all nurses working in any area of discipline should possess the skills to deliver general EOL care, which includes physical symptom management, psychosocial support and discussions about goals of care and prognosis. This is especially important as EOL is a complex process where a patient's condition may decline rapidly, leading to emotionally challenging situations that require professionalism, clinical knowledge and maturity (Fristedt et al. 2021).

Nursing students worldwide described feeling poorly prepared to care for patients at EOL (Yoong et al. 2023). This feeling extends to their first year of practice following their studies. Croxon et al. (2018) found that many nursing graduates did not feel ready to care for people at EOL, with a key theme being graduates feeling poorly equipped to communicate with families and carers. Similarly, another study exploring the experiences of graduate nurses when caring for patients at EOL identified the need for the graduate to feel confident and prepared to implement quality and safe care (Deravin‐Malone et al. 2016).

One teaching and learning method which has been used in nursing education to support students integrate theory to clinical practice is simulation‐based education (SBE). According to Gaba (2007), a founding and international leader in simulation, SBE is described as ‘a technique—not a technology—to replace or amplify real experiences with guided experiences that evoke or replicate substantial aspects of the real world in a fully interactive manner’ (126). Simulation in undergraduate nursing education is an innovative pedagogic approach that enhances critical thinking, clinical reasoning, problem‐solving and communication (Parry et al. 2022). Simulation has been demonstrated to help students link EOL theory to practice, improving their assessment skills in EOL clinical situations (Aamlid and Tveit 2022). Given the benefits to student learning and clinical practice from participating in EOL simulation, mapping the use, design and facilitation approaches of simulation to educate undergraduate nursing students in EOL care is warranted.

## 2 Background

Up to 50% of deaths worldwide occur in acute hospital settings (Witkamp Frederika et al. 2015), with literature stating hospitals in the future will become the default setting where expected deaths occur (Rawlings et al. 2020). There is an increasing number of patient deaths in acute hospitals, and no guarantee all patients will be admitted to a dedicated palliative care ward (Croxon et al. 2018); therefore, all health professionals need to feel confident and prepared to provide safe and quality EOL care.

Current literature identifies that EOL education across many countries, including Australia, lacks the depth and consistency (Nilsson et al. 2022; Pereira et al. 2021) to adequately prepare students for the workforce (Gillan et al. 2014). Some European countries do not view palliative care education as a mandatory topic in the undergraduate nursing curriculum (Pereira et al. 2021) and there is limited evidence of EOL education being implemented in the undergraduate nursing curriculum in the US (Condry and Aucoin 2024). Mastroianni et al. (2021) reason that the education provided is mainly theoretical with minimal opportunities to integrate learning into clinical practice.

Research has demonstrated that simulation as a learning strategy in EOL education can enhance student knowledge, confidence, attitude and communication skills (Hoang et al. 2022). The use of standardised patients (SPs) in EOL simulations, where a person plays a role to facilitate learning (Hall et al. 2024), has also been shown to develop similar attributes in students (Çakmak and İnkaya 2024). Additionally, virtual reality simulation, which utilises computer‐generated simulations, has revealed similar benefits, particularly in student confidence and communication in EOL care (Hall et al. 2024). Overall, students evaluated EOL simulations as a positive learning activity without the fear of consequences of errors (Bassah et al. 2014), with some students requesting additional EOL simulations to support their learning (Heise et al. 2018).

Research has identified some important findings for consideration when integrating EOL simulation in undergraduate nursing curricula. Designing and clearly articulating intended learning outcomes and content topics during the EOL simulation experience contributes to a positive learning experience for students (Aamlid and Tveit 2022). Additionally, the role of the facilitator in EOL simulations has been highlighted as a crucial component of the student learning experience and in supporting a psychologically safe learning environment (Hoang et al. 2022). Bloomfield et al. (2015) indicate that EOL simulations decrease student stress and anxiety levels as they provide enhanced preparation for the reality of clinical practice. In another study, students reported that although EOL simulations led to heightened emotional impact, they were beneficial for their learning and developing communication skills (Ruiz‐Pellón et al. 2020).

The benefits of EOL simulations to undergraduate nursing education and practice are significant. However, there is limited research worldwide and in Australia investigating and reporting on EOL simulations in undergraduate nursing curricula. An integrative review by Condry and Kirkpatrick (2021) identified 11 articles related to EOL simulations in undergraduate nursing curricula from countries including the United States, Norway, Spain, Japan and the United Kingdom (Condry and Kirkpatrick 2021), highlighting the limited reporting of EOL simulations embedded in curricula. It is therefore important to map EOL simulation integrated into undergraduate nursing curricula to identify how EOL simulations have been used and explore the quality of these SBE experiences.

## 3 The Study

The aim of this study was to explore EOL simulation in undergraduate nursing curricula in Australian and New Zealand institutions.

## 4 Research Question

The research questions posed in this study included:

What EOL SBE is currently incorporated in the undergraduate nursing curricula in Australia and New Zealand?

- What simulation modalities and components are used in EOL simulations?
- What preparations are required for facilitators to deliver EOL simulations?

## 5 Methods

This study was conducted as part of a larger research project underpinned by a critical realist philosophical stance. The aim of critical realism is to determine which causal mechanisms are important and what effect they have (Stutchbury 2021). This study reports on the empirical elements of the phenomenon, that which is seen or can be measured: identifying what EOL education experiences using simulations are integrated in undergraduate nursing curricula in Australia and New Zealand. The Consensus‐Based Checklist for Reporting Survey Studies (CROSS) checklist (Sharma et al. 2021) (File S2) was used to report the findings of this study.

### 5.1 Research Design

This study used a cross‐sectional descriptive research design involving data collection from an online survey of nursing education institutions in Australia and New Zealand. In Australia and New Zealand, nursing curricula are governed by accreditation organisations including the Australian Nursing and Midwifery Council (ANMAC) and the Nursing Council of New Zealand. Curricula are updated every 5 years as part of the process of updating and reviewing the education programme accreditation. Therefore, a cross‐sectional design captured the status of EOL simulations in the undergraduate nursing curricula in Australia and New Zealand in 2023.

### 5.2 Sampling and Recruitment

All institutions with an undergraduate bachelor's degree in nursing leading to registration as a registered nurse with the Australian Health Practitioner Regulation Agency or the Nursing Council of New Zealand were eligible for inclusion. Institutions were identified through the Council of Deans of Nursing and Midwifery (Australia and New Zealand) (CDNM) website. The CDNM was contacted, asking them to forward a letter of invitation to heads of schools or departments. The letter requested heads to identify either a coordinator of the subject in which EOL care is taught, or a coordinator of the undergraduate nursing programme to complete the survey. Additionally, course coordinators of Bachelor of Nursing programmes were identified via institution web pages and were directly emailed using publicly available contact details. Forty‐five institutions (38 from Australia, 7 from New Zealand) with accredited Bachelor of Nursing programmes were invited to participate.

### 5.3 Instrument and Data Collection

Using a set of key reporting elements for simulation‐based research (Cheng et al. 2016), an online instrument was developed for this study (File S1). Once developed, the instrument underwent testing for face validity by the research team prior to use. Content validity was assessed by two independent faculty who were deemed experts in the field of simulation. The reviewers were asked to consider if any important aspects of the instrument related to simulation had been omitted and if any language used was unclear. Information technology experts also reviewed the flow and logic of the instrument. No content was deemed missing, although some wording suggestions were made to improve readability and understanding of survey questions.

Definitions of the terms used in the instrument were adapted from the International Nursing Association of Clinical Simulation and Learning (INACSL) Standards of Best Practice: Simulation Glossary (INACSL Standards Committee 2016a). The survey comprised eight domains: simulation orientation, simulator type, simulation environment, instructional design, simulation event, pre‐brief, debrief and facilitation preparation and requirements. Including demographic questions, the survey included 66 questions and was estimated to take 20 min to complete. The survey was presented and collected on QuestionPro, a research survey software, between January and September 2023.

### 5.4 Data Analysis

Raw data were downloaded into Microsoft Excel and each respondent was given an anonymous unique identifier. Prior to analysis, data were cleaned by inspecting each response for completeness. Any survey responses which did not complete past the demographic questions were deemed ineligible for analysis and discarded. Five responses were discarded following this review. Next, the researchers inspected each survey response, investigating whether the questions had been understood by the participants. Extreme outliers and accuracy of the data were assessed. No data were excluded because of this assessment.

Following data cleaning, both demographic and survey data were analysed using descriptive statistics. Categorical variables were presented as counts and percentages. First, the demographics of responses describing the institutions and the presence of EOL simulation in the undergraduate curricula are presented. Second, reports on the inclusion of EOL simulations in the curriculum are presented. Third, due to the large number of questions asked in the survey, to assist in reporting the results, data are reported under domains of the instrument: simulation orientation, simulator type, simulation environment, instructional design, simulation event, pre‐brief, debrief and facilitation preparation and requirements.

### 5.5 Ethical Considerations

Ethics approval was granted by the Human Research Committee. Consent was obtained from participants using a tick‐box consent button at the beginning of the survey. All participants who undertook the survey remained anonymous and non‐identifiable. Participants were provided with an option to identify their institution if they wanted to; to prevent re‐identification of institutions, only aggregate data were presented. Data are stored on the QuestionPro server and on a secured institutional server. Both servers remain password‐protected and only researchers have access to the data. Data will be stored in line with the University Research Data Management Policy.

## 6 Results

### 6.1 Demographics

Responses were received from 30 institutions, *n* = 21 were from Australia and *n* = 9 were from New Zealand. Twenty‐five surveys were sufficiently complete to analyse, *n* = 20 from Australia (*n* = 7 from New South Wales, *n* = 1 from South Australia, *n* = 1 from Tasmania, *n* = 10 from Victoria, and *n* = 1 from Western Australia) and *n* = 5 from New Zealand. Eleven institutions responded yes to including EOL simulations in their undergraduate nursing curriculum, of which *n* = 10 were from Australia (*n* = 4 from New South Wales, *n* = 5 from Victoria, and *n* = 1 from Western Australia), and *n* = 1 was from New Zealand. One institution reported having previously had EOL simulations in their undergraduate curriculum, and one reported planning to include EOL simulation in future curricula.

### 6.2 Inclusion of EOL Simulations

Of the 11 institutions which reported having EOL simulation in their curriculum, simulations focussing on EOL care had been embedded into the undergraduate nursing curriculum for between one and 8 years. The total hours spent teaching with EOL simulations ranged from 1 to 6 h. EOL simulations were reported to be taught across all year levels, but predominantly in the third year of the programme (*n* = 6). One institution reported embedding three different EOL simulation scenarios in their curriculum across years one, two and three.

### 6.3 Simulation Orientation

All institutions (*n* = 11) reported providing an orientation for students to the simulation environment and the simulator prior to the simulation.

### 6.4 Simulator Type

Simulation modalities varied across institutions and included high fidelity full body manikins (*n* = 4), SPs (*n* = 3), low‐fidelity full‐body manikins (*n* = 3), part‐task trainers (*n* = 1) and virtual reality patients (*n* = 1). The styles used to facilitate EOL simulations included immersive (*n* = 8), fishbowl (*n* = 5) and tag team (*n* = 2). Pause and discuss, and observational styles of facilitation were not used. Five institutions reported the use of SPs in the role of either the patient (*n* = 3) or family member (*n* = 2).

### 6.5 Simulation Environment

**FIGURE 1:**
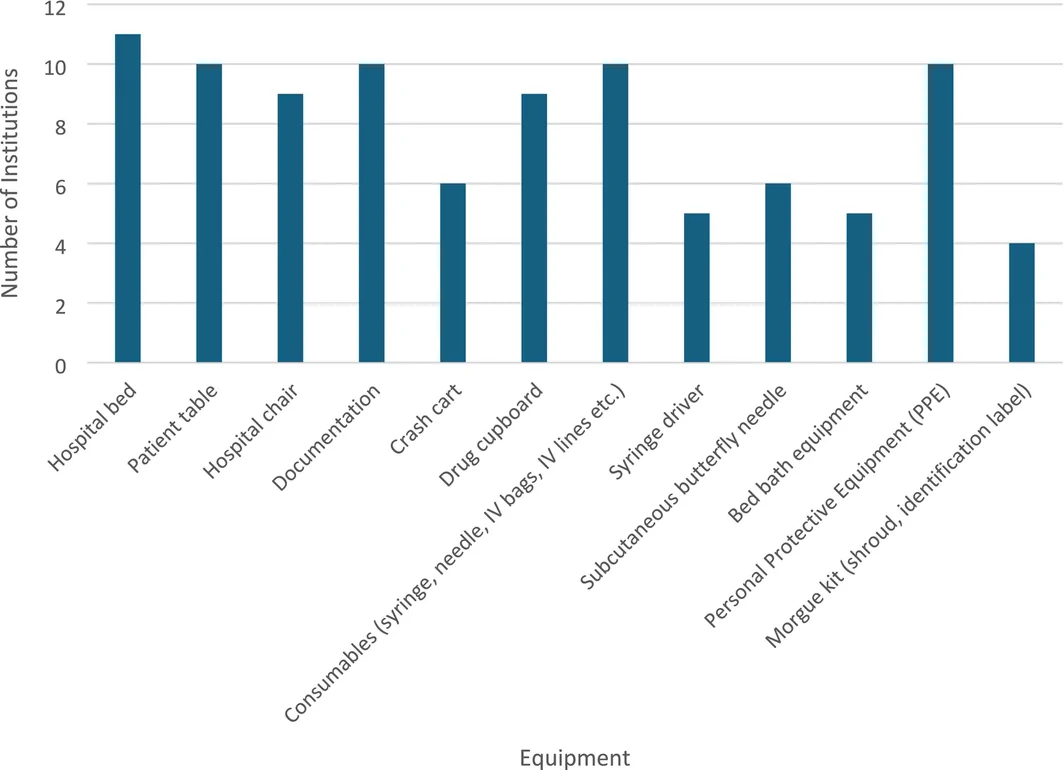
Equipment available to students for the EOL simulation. [Colour figure can be viewed at wileyonlinelibrary.com]

The learning environment where EOL simulations were conducted included dedicated simulation rooms with a control room (*n* = 6), of which four had a dedicated observation room. Other learning environments reported were clinical lab rooms that students also used to learn practical skills (*n* = 5) or classrooms (*n* = 1). While the learning environment differed between institutions, all EOL simulations had at least a hospital bed available to students. Other equipment available in the learning environment varied across institutions (Figure 1).

### 6.6 Instructional Design

All institutions reported providing students with EOL education prior to the simulation, such as lecture material (*n* = 5), classroom‐based teaching (*n* = 6), lab‐room‐based teaching (*n* = 5) or self‐directed learning (*n* = 11). These preparations were provided to students either one to 2 weeks prior to engaging in the simulation (*n* = 7), or immediately prior to the simulation (*n* = 1), or both (*n* = 2).

Three institutions reported designing their simulation into frames or segments while eight did not. Simulations were designed to run for various durations. The three institutions that reported using frames reported each frame ranging from 1 to 20 min. The total duration of the simulation was between 21 min and 4 h. The eight institutions that reported no use of frames had simulation durations ranging from 11 min to 4 h.

Two respondents reported allowing participants the opportunity to repeat the EOL simulation during the same scheduled session. One respondent reported allowing one repetition, and the other reported three repetitions in one scheduled simulation session. No institutions repeated the EOL simulation later. Respondents who did not allow repetition qualified that their students were offered an opportunity to repeat learned skills in clinical skills practice sessions to enhance learning, while others (*n* = 2) reported repetition was not supported due to lack of time or space.

All but one institution (*n* = 10) used simulation guidelines to help in the development and design of their EOL simulation, with the simulation guidelines varying across institutions. Most opted to use university‐developed guidelines (*n* = 8), while others adapted guidelines from INACSL (*n* = 2) or the Palliative Care Curriculum for Undergraduates (PCC4U 2020) (*n* = 3). The one institution that did not use a guideline reported that the development and review of the EOL simulation were undertaken by nursing academics within the institution with expertise in simulation.

Eight institutions reported using standardised templates to assist in the design of their EOL simulation. Following the completion of the scenario design, 10 respondents reported peer reviewing their EOL simulation, and eight reported pilot testing the EOL simulation.

### 6.7 Simulation Event

Eight institutions provided a script to the person who assumed the role of the patient, while three did not. Seven institutions provided a script to guide confederates (secondary person who assumes a role to support simulation facilitation, e.g.,: registered nurses or medical practitioners), while four did not. Lastly, six institutions provided a script to support family members, while five did not. Of the five institutions that did not provide a script to family members, four reported providing no preparation at all, while one institution reported providing a separate pre‐brief as well as a verbal orientation to support the role.

**FIGURE 2:**
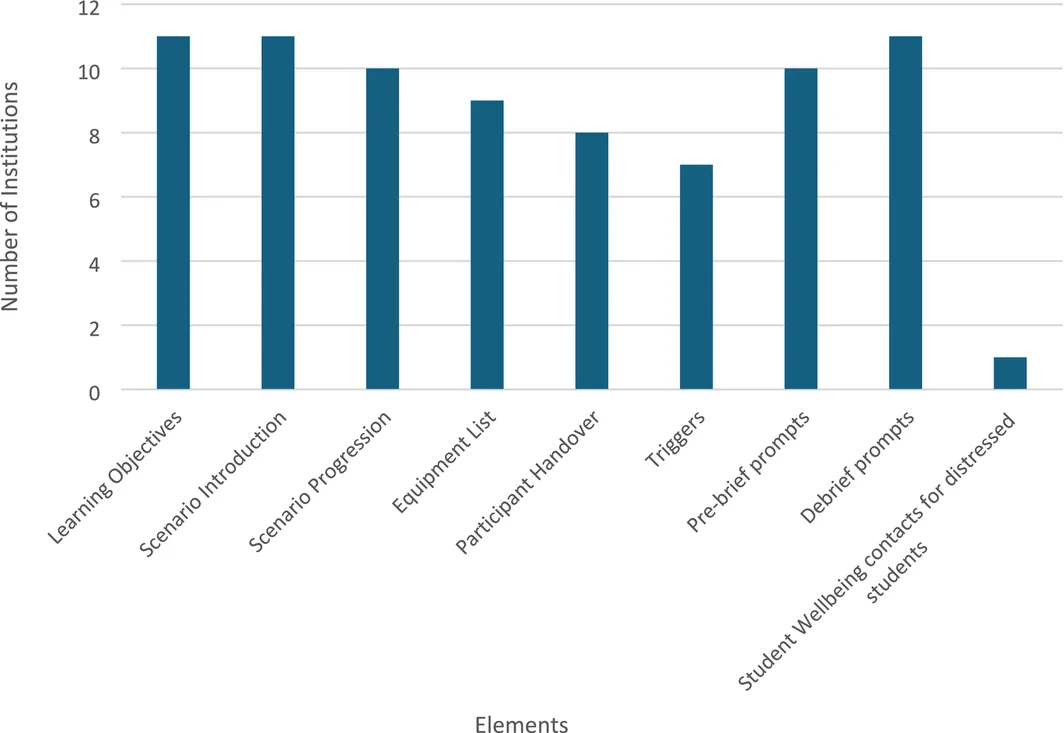
Elements included in the instructor's guide. [Colour figure can be viewed at wileyonlinelibrary.com]

All institutions provided faculty with a simulation instructor guide to assist in facilitating the EOL simulation, which included information listed in Figure 2. Only one institution reported including contact information for student well‐being in case students became distressed.

**FIGURE 3:**
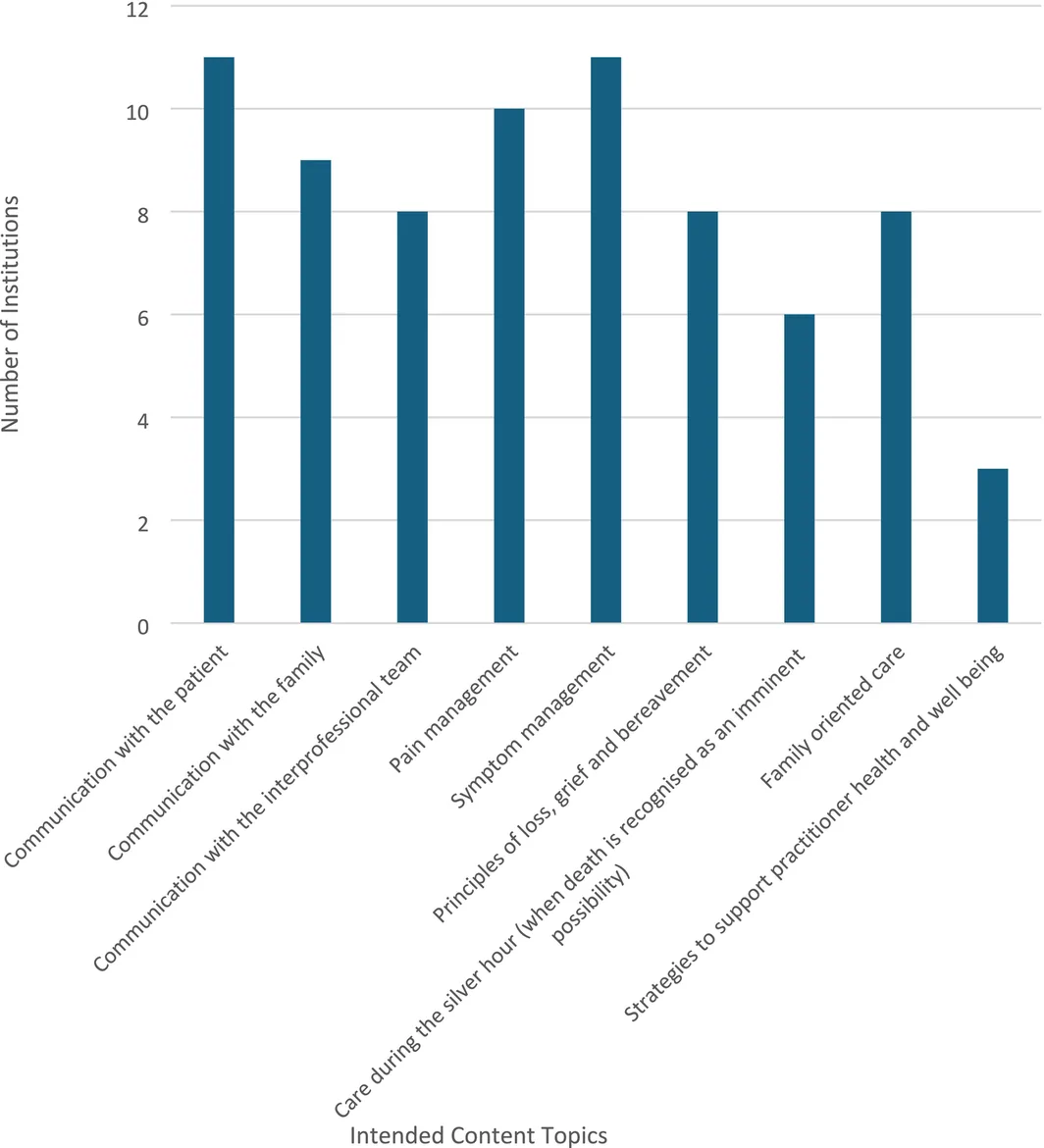
Intended content topics for the EOL simulation. [Colour figure can be viewed at wileyonlinelibrary.com]

The intended content topics for EOL simulations differed across institutions as reported in Figure 3

Of those institutions that reported using segments, the number of students participating in each segment varied between one and four. Respondents who reported not having segments or frames in their simulation had up to 15 students participating at one time. One institution reported running a simulation with 90 student participants present in one frame, and another reported between 25 and 30 students.

In addition, many institutions designed the EOL simulation scenario to include other roles, such as family member (*n* = 6), doctor (*n* = 4) and chaplain (*n* = 1). No institutions reported having allied health roles included in their EOL simulation. Six institutions reported including the role of a family member in their EOL simulation. This role was played by either faculty (*n* = 3), SPs (*n* = 2) or a student (*n* = 1). Students were allocated roles such as student nurse (*n* = 7), registered nurse (*n* = 6), doctor (*n* = 1) or patient (*n* = 1).

### 6.8 Pre‐Briefing

**FIGURE 4:**
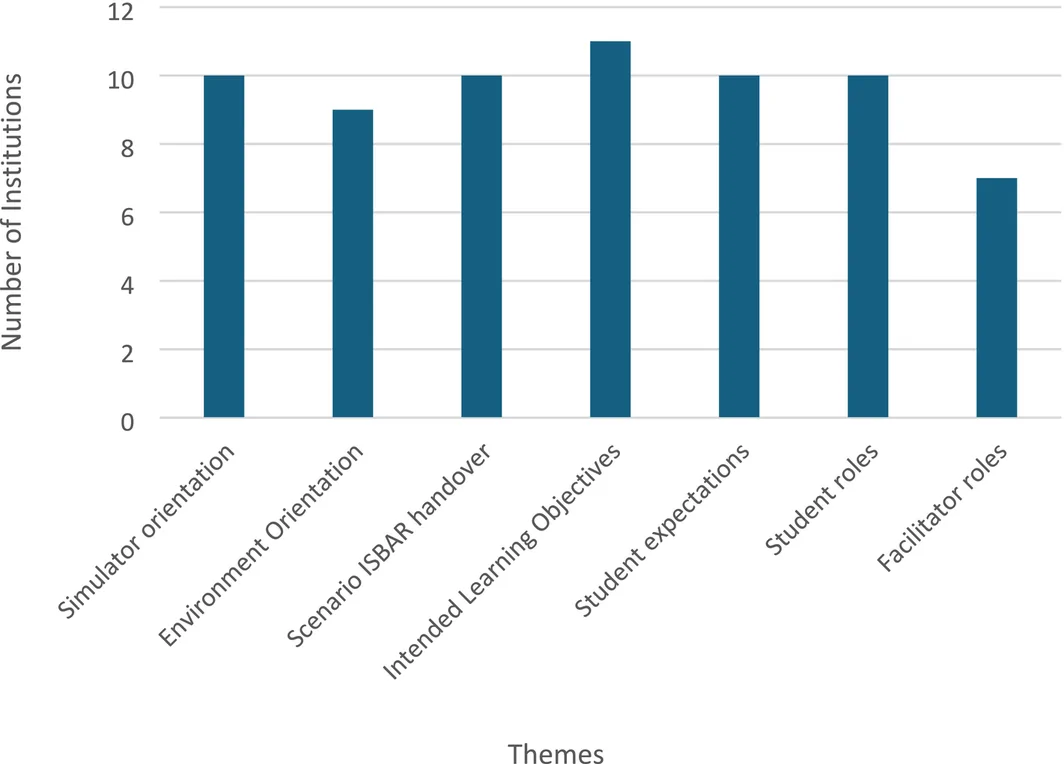
Themes discussed in EOL simulation pre‐briefing. [Colour figure can be viewed at wileyonlinelibrary.com]

All respondents reported pre‐briefing students prior to entering their EOL simulation. The pre‐briefing ranged from 1 to 50 min. The themes discussed in the pre‐briefing by each institution are shown in Figure 4. Six respondents reported their EOL simulations led to simulator death, with two respondents informing students the simulator would die in the scenario. While facets of psychological safety for students were not listed in the survey, one of the two institutions included a free text comment reporting this was addressed in the pre‐briefing.

### 6.9 Debriefing

All respondents reported debriefing students following their EOL simulation. Two institutions reported debriefing took place after each segment and at the end of the entire simulation, while in nine, it occurred only at the end of the entire simulation. Debriefing ranged from 1 to 60 min. The debrief model most often used was the Patient Experience and Reflective Learning (PEARLS) reflection model (Eppich and Cheng 2015) (*n* = 5), followed by the Debriefing for Meaningful Learning Model (Dreifuerst 2015) (*n* = 4). Other models used by institutions included the Plus‐Delta Model (Cheng et al. 2021) (*n* = 2), the Advocacy/Inquiry Model (Rudolph et al. 2007) (*n* = 1), the Structured Interview Guide (*n* = 3) and the Diamond Debriefing Model (Jaye et al. 2015) (*n* = 1). Eight respondents reported a script was provided for faculty to assist with debriefing. All respondents reported offering counselling services to students during the debrief.

### 6.10 Facilitation Preparation and Requirements

Institutions reported having one (*n* = 4), two (*n* = 5) or more than two (*n* = 2) simulation educators facilitating the EOL simulation. Eight institutions reported having simulation technicians assist with the use of simulators; four of these ran the simulation with one educator.

The facilitation of the EOL simulations was performed by nursing academic faculty (*n* = 8), casual faculty (*n* = 8), or clinical educators (*n* = 4). No EOL simulations were facilitated by either medical or allied health professionals. One institution reported facilitators of the EOL simulation in their program required a master's level qualification in palliative care. Ten respondents stated that facilitators of EOL simulations were not required to have any postgraduate qualification in palliative care. Furthermore, nine institutions reported no requirement for facilitators of EOL simulation to have clinical expertise in palliative care nursing, while two required clinical expertise.

Ten institutions reported preparing faculty to facilitate the simulation using a variety of different measures. To assist in faculty preparedness to facilitate simulations in general, respondents reported running either simulation study days (*n* = 9), having online simulation modules (*n* = 4), or using a supernumerary (shadowing a more senior simulation facilitator) model (*n* = 1). Regarding EOL content preparation, institutions reported offering online training modules (*n* = 2), study days (*n* = 4), information packages (*n* = 9) or simulation run‐throughs (without students present) (*n* = 1). Some institutions adopted more than one standard preparation model.

## 7 Discussion

This study is the first to our knowledge to report on EOL simulations in undergraduate nursing curricula in Australia and New Zealand. The findings of the study suggest that there are variations in what EOL SBE is integrated into the curriculum, including modalities used and preparations required for facilitators. The EOL simulations reported comply with the Professional Development, Outcomes and Objectives, and The Debriefing Process INACSL Healthcare Simulation Standards of Best Practice. However, there are inconsistencies between institutions in their practices with Pre‐briefing and Facilitation Methods.

Eleven institutions reported integrating EOL simulations into their curricula. Given the reported benefits of the EOL simulation outlined above, the lower than anticipated rate of integration of EOL simulation in curricula is surprising. However, the lack of consistency in the use of EOL simulation to prepare students for EOL care in practice may result from the lack of standardisation for EOL care education in undergraduate nursing curricula in Australia and New Zealand (ANMAC 2019; Nursing Council of New Zealand 2022). In Australia and New Zealand, undergraduate nursing curricula are governed by the ANMAC, or the Nursing Council of New Zealand, which stipulates that programme content and learning outcomes should ensure ‘integrated knowledge of care across the lifespan and across contexts of nursing practice’ (ANMAC 2019, 12) but do not stipulate topics or teaching and learning approaches. Hence, for those institutions that have embedded palliative and EOL care education into the curriculum, there is no streamlined approach or time stipulated to teach the content (Hegarty et al. 2010). Although this study has highlighted 11 institutions that have embedded EOL simulations into their curriculum, it does not consider other EOL content available. Therefore, further investigation to explore why some institutions choose to include or not include EOL simulation in undergraduate nursing curricula is warranted.

### 7.1 Professional Development

All institutions provided professional development regarding the pedagogy and facilitation of a simulation to ensure staff were able to facilitate a quality simulation‐based learning experience for students. The results of this study identified professional development provided to facilitators centred around general simulation facilitation and EOL‐specific content. This need for initial and ongoing professional development for facilitators in simulation is reflected in the development of the Healthcare simulation standards of best practice professional development (Hallmark et al. 2021). Prior to this standard, there was less focus on facilitator competencies and greater emphasis on participant outcomes (Cheng et al. 2015). Authors have identified the impact of facilitators' skills on the educational outcomes of simulation (Poore et al. 2022), such as communication, creating a psychologically safe learning environment, maintaining simulation fidelity and understanding pedagogical principles that support simulation (Madsgaard et al. 2022). While this study found that some professional development is offered to facilitators, it did not identify whether it was provided as a single event or was ongoing. This may be an area that merits further investigation.

### 7.2 Facilitation

The skills, experiences, and knowledge of the academic faculty facilitating the simulation varied. The findings from this study identified only one institution that required facilitators to have undertaken further studies leading to a qualification in palliative care to teach EOL simulations. There is a lack of consensus between institutions on who should facilitate these EOL simulations and what educational preparation and clinical expertise they should have, which is reflected in the literature. Yoong et al. (2023) suggested clinical experts in palliative care should be involved in the facilitation of EOL simulations. Similarly, Gillan et al. (2014) argued that faculty who facilitate EOL simulations should be adequately qualified and trained in simulation to support learner needs. Juvet et al. (2023) and Üzar‐Özçetin et al. (2021) have aligned viewpoints in that they advocate simulation facilitators should be subject matter experts. Josephsen and Martz (2014) found facilitators often have a knowledge deficit when teaching EOL care in general, making it a barrier to teaching. On the other hand, McGaghie et al. (2010) contend that clinical experience in a field does not assist in the effective facilitation of a simulation. In a recent study, Díaz et al. (2020) revealed there was no notable difference in the effectiveness of a simulation facilitated by a content expert. Despite the inconsistencies, authors suggest that faculty's knowledge on the topic, communication and ability to establish a safe learning environment have been identified as important attributes that promote a positive simulation experience for students (INACSL Standards Committee 2016b; Aamlid and Tveit 2022; Hoang et al. 2022). The influence of the facilitator's experiences, expertise in, and knowledge of simulation facilitation and of palliative and EOL care on student learning experiences during EOL simulations warrants further investigation.

### 7.3 Simulation Design

The findings of this study suggest that there is a purposeful simulation design that uses experts in simulation, combined with simulations that are developed predominantly using templates, which were peer‐reviewed and pilot‐tested before being implemented with students. Moreover, each EOL simulation had learning objectives of the EOL simulation documented in the facilitator's guide and identified to students in the pre‐brief.

In this study, the dominant simulation modality was high fidelity full body manikins, followed by using SPs. Authors argue that the use of SPs in EOL simulations has been demonstrated to increase the self‐efficacy, knowledge, and communication skills of students in EOL care (Çakmak and İnkaya 2024). Similarly, Escribano et al. (2021) found the use of SPs in EOL simulations improved communication self‐efficacy, empathy, respect and assertiveness of students. A study comparing the use of SPs and manikins in mental health simulations found the use of SPs resulted in greater realism, improving communication, safety and the student's own confidence (Luebbert et al. 2023). However, a recent scoping review identified challenges in using SPs for nursing simulations, which included a lack of psychological safety for SPs, absence of policies and guidelines related to the use of SPs, and limited pre‐briefing and debriefing processes that incorporate the use of SPs (Bozkurt et al. 2023). Additional research is needed to explore the role and influence of SPs on student learning experiences and outcomes in EOL simulations and identify any barriers and enablers given the benefits realised.

### 7.4 Pre‐Briefing

McDermott et al. (2021) identify pre‐briefing as an important aspect of SBE, including establishing psychological safety, preparing for learning in the simulation, and conveying the ground rules for simulation. Additionally, a recent study by Solli et al. (2020) highlights the need for expertise in pre‐briefing as it is viewed as a demanding and complex role whereby the facilitators balance the diverse needs of learners. Pre‐briefing has now been established as just as important as debriefing (Chamberlain 2016). The results of this study identified inconsistencies around time spent on the pre‐brief and discrepancies with the themes covered in the pre‐brief (Figure 4). While the results of this study do not talk about the effectiveness of pre‐briefing, the findings suggest there is not one structured model for pre‐briefing that is used across institutions. This aligns with the views that identifying the appropriate pedagogies for pre‐briefing remains a challenge (Chan et al. 2023; McDermott et al. 2021).

Pre‐briefing activities assist in the development of a psychologically safe learning environment and involve mentally preparing the learners around the educational content, as well as communicating the ground rules for the simulation (Chan et al. 2023; McDermott et al. 2021). Creating a psychologically safe environment is especially important when the topic of the simulation is sensitive, such as EOL care. It can be argued that informing students about simulator death may help in promoting psychological safety. Alinier and Oriot (2022) contend that concealing some aspects or content of the scenario can provide learners with a more authentic experience; however, this is generally dependent on the student's level of experience and the intended learning outcomes of the simulation. In an EOL simulation for undergraduate learners, facilitators need to prepare students by informing them that their simulator will die (Corvetto and Taekman 2013; Gaba 2013). This study showed two institutions reported informing students that the simulator would die. The link between the psychological safety of students and the information that the patient in the scenario may die warrants further investigation.

### 7.5 Debriefing

Nunes and Harder (2019) stress the significance of a debriefer who is knowledgeable of the content and can maintain a psychologically safe learning environment. At the completion of the EOL simulation scenario, a planned debrief with the use of a debriefing model was conducted by all institutions. Some institutions provided an additional script to assist facilitators with the debriefing process. In addition, all institutions reported offering access to counselling services to students during the debrief. The debriefing process is fundamental in SBE activities as it can help students process any emotions induced by the simulation and improve learning (Jablonski et al. 2020). In EOL simulations, debriefing can help students feel prepared to cope with their emotions in practice and develop healthy coping mechanisms (Nunes and Harder 2019). This study did not report on whether any debriefing model generated greater reflection or exploration of knowledge. This may be an area of interest for further research.

## 8 Strengths and Limitations

The strengths of the study lie in that it adhered to the guidelines for conducting a cross‐sectional survey, ensuring methodological rigour. In addition, data were collected from a diverse range of institutions in Australia and New Zealand, rather than a single site, strengthening the potential to generalise the findings to a broader population.

However, there are limitations that must be acknowledged. The current study had an inclusion criterion to capture all 45 institutions in Australia and New Zealand with an undergraduate nursing degree. However, only 30 institutions responded, which means the data collected may not fully represent the population. The study can be subject to sampling and reporting bias as the survey was only completed by those who chose to participate. Despite this limitation, the strengths, which include the broad geographic scope and methodological standards, provide valuable insight for future investigations.

## 9 Recommendations for Future Research

This study has identified a small number of institutions in Australia and New Zealand that have embedded EOL simulations in their undergraduate curriculum. The reason why this is the case is unclear and would be an area for further research. Furthermore, there are variations in the design and facilitation of EOL simulations in undergraduate nursing programs. Further research is recommended to investigate the learning experiences and outcomes of students in various modalities of EOL simulations. Pre‐briefing, particularly regarding whether students should be informed about the simulator's expected death and consequences, with and without this information, requires further clarity. The optimal qualifications and content expertise of simulation facilitators, and approaches used to facilitate EOL simulation and the influence this has on the student learning experience require further research.

## 10 Conclusion

End‐of‐Life simulations are integrated into 11 undergraduate nursing curricula across Australia and New Zealand. Importantly, pre‐briefing and debriefing are reported to be conducted across all institutions that integrate EOL simulation to support student learning. Most of the EOL simulations are designed using the Healthcare Simulation Standards of Best Practice (Watts et al. 2021).

However, this study identified that while EOL simulation is integrated into undergraduate nursing curricula, there are differences in the modality and approaches used. While SPs are suggested to have significant benefits for student learning, they are not the dominant modality used in EOL simulation in undergraduate nursing. Further research to explore the barriers and enablers to using SPs in EOL simulation in undergraduate nursing education is needed. Additionally, while pre‐briefing appears to be consistently conducted across the institutions that use EOL simulation, the elements to be covered and appropriate pedagogy around this pre‐briefing for undergraduate nursing students lack clarity. Moreover, the role and influence of different debrief models following EOL simulation in undergraduate nursing on student learning and preparation for practice require further investigation.

Finally, evidence suggests that while professional development is provided to simulation facilitators, the requirements regarding clinical expertise and knowledge and experience of faculty to facilitate simulation vary. How the qualifications, expertise and experiences of the EOL simulation instructors influence the facilitation and the learning experiences of participants in the EOL simulation warrants additional research.

## Conflicts of Interest

The authors declare no conflicts of interest.

## Supporting information


Appendix S1.

Appendix S2.

## Data Availability

The data that support the results of this study are not available as data sharing has not been approved by the Human Research Ethics Committee.
