# Supplementary material for: End‐Of‐Life Simulation in Undergraduate Nursing Curricula: A Cross‐Sectional Survey
Source: J Clin Nurs. 2025 Apr 11;35(10):4273–84. doi: 10.1111/jocn.17776 (PMC13569350; doi:10.1111/jocn.17776)
Supplement: Supplementary file 2 [file JOCN-35-4273-s002.doc]

**Introduction**

Thank you for your interest in participating in the "End-of-life (EOL) Simulations in Australian and New Zealand Undergraduate Nursing Curricula” survey.

For the purpose of this survey, the INACSL Standards of Best Practice: Simulation Glossary will be used.

Simulation is defined as "an educational strategy in which a particular set of conditions are created or replicated to resemble authentic situations that are possible in real life" (INACSL Standards Committee, 2021, p. 62).

Additional information about the study is included in the Participant Information Statement and Consent Form.

Please click "Start" to provide your consent and begin the survey. If you have questions at any time about the survey or the procedures, you may contact Cindy Hoang at 03 9479 5956 or by email at the email address specified below.

Thank you very much for your time and support.

Regards

Cindy Hoang

[c.hoang@latrobe.edu.au](mailto:c.hoang@latrobe.edu.au)

**Consent**

I have read the information presented to me, and any questions have been answered to my satisfaction. By completing this survey, I agree to participate in the study. I know I can withdraw my data at any time prior to data analysis, without penalty. I agree information provided by me or with my permission during the project may be included in a thesis, presentation and published in journals on the condition that I cannot be identified.

1. Yes
2. No

**Demographic Information**

Institution Name (optional):

Academic Position (optional):

Country of Residence

1. Australia
2. New Zealand

State:

1. Australian Capital Territory
2. New South Wales
3. Northern Territory
4. Queensland
5. South Australia
6. Tasmania
7. Victoria
8. Western Australia

Do you have EOL simulation in your undergraduate nursing curriculum?

1. Yes
2. No **<survey ends>**
3. No, but we used to **<survey ends>**
4. No, but we plan to **<survey ends>**

How many EOL simulations are embedded in your curriculum?

1. 1
2. 2
3. 3
4. >4

Which year is EOL simulation embedded into your curriculum? (Tick all that apply)

1. 1st year
2. 2nd year
3. 3rd year
4. 4th year

How many years has EOL simulation been in your curriculum?

1. 1-2 years
2. 3-4 years
3. 5-6 years
4. > 6 years

How many hours of EOL simulation education do your students receive?

1. 1-2 hours
2. 3-4 hours
3. 5-6 hours
4. > 6 hours

What is the average number of students per EOL simulation session?

1. < 10 students
2. 10 - 15 students
3. 16 - 20 students
4. > 20 students

In the last year, how many facilitators/educators, in total, did you have teaching into EOL simulation in your course?

1. < 5
2. 6 - 10
3. 11 - 15
4. > 15

How many facilitators/educators do you have teaching into each EOL simulation session?

1. 1
2. 2
3. 3
4. > 3

Do you have a simulation technician present to assist you with running the EOL simulation?

1. Yes
2. No

**Simulation Orientation**

Are students provided an orientation to the simulator?

1. Yes
2. No

Are students provided an orientation to the simulation environment?

1. Yes
2. No

**Simulator Type**

What simulation modality is used to teach EOL simulation? (tick all that apply)

1. Part task trainer (partial manikin to simulate a specific task/procedure/skill)
2. Low fidelity full body manikin (low technological ability to mimic human body functions)
3. High Fidelity full body manikin (high technological ability to mimic human body functions)
4. Standardised patient (human actors)
5. Computerised virtual patients (computer technology is used to create an on-screen virtual patient)
6. Hybrid (using two different modalities in one scenario)
7. Other <comment box provided>

What simulation facilitation style is used to teach EOL simulation? (tick all that apply)

1. Fishbowl (a number of learners are active participants in the simulation while observers stand in a fishbowl around them)
2. Pause and discuss (deliberate pause and discussion points held during the simulation)
3. Tag team (learners tag in and out of a simulation scenario, assuming the same role as the previous active participant)
4. Immersive (learners enter a recreation of real-world scenario that allows the scenario to play out until the end with no interruptions)
5. Observational (learners take a purely observational stance)
6. Other <comment box provided>

If actors are used in EOL simulation, what is their role? (tick all that apply)

1. Patient
2. Family
3. NA

**Simulation Environment**

Where is the EOL simulation conducted? (tick all that apply)

1. Simulation room
2. Lab room
3. In situ clinical environment
4. Classroom
5. Other <comment box provided>

Is there a dedicated control room?

1. Yes
2. No

Is there a dedicated observation room?

1. Yes
2. No

What equipment is available to students for the EOL simulation? (tick all that apply)

1. Hospital bed
2. Patient table
3. Hospital chair
4. Documentation
5. Crash cart
6. Drug cupboard
7. Consumables (syringe, needle, IV bags, IV lines etc.)
8. Syringe driver
9. Subcutaneous butterfly needle
10. Bed bath equipment
11. Personal Protective Equipment (PPE)
12. Morgue kit (shroud, identification label)
13. Other <comment box provided>

**Instructional Design**

Are there any non-simulation interventions on EOL education prior to the simulation? (e.g.: lecture, tutorial)

1. Yes
2. No

What non-simulation interventions are used? (Tick all that apply)

1. Lecture
2. Classroom based
3. Lab class
4. Self-directed learning
5. Other <comment box provided>

When are non-simulation interventions used relative to the simulation? (Tick all that apply)

1. Immediately prior to simulation
2. 1-2 weeks prior to simulation
3. Same semester as simulation
4. Other <comment box provided>

What is the total duration of the EOL simulation scenario?

1. 0-10 minutes
2. 11-20 minutes
3. 12-30 minutes
4. > 30 minutes

Does the EOL simulation involve segments/frames?

1. Yes
2. No <skip next question>

What is the duration of each EOL segment/frame?

1. 0-10 minutes
2. 11 - 20 minutes
3. 21 - 30 minutes
4. > 30 minutes

Do students have an opportunity to repeat the EOL simulation in the same session?

1. Yes
2. No <skip next question>

How many times is repetition of the EOL simulation permitted?

1. 1
2. 2
3. 3
4. > 3

Do students have an opportunity to repeat the EOL simulation at a later time?

1. Yes
2. No <skip next question>

When are students given the opportunity to repeat the EOL simulation at a later time?

1. The same day
2. The same week
3. The same semester
4. Other <comment box provided>

How many times is repetition of the EOL simulation permitted?

1. 1
2. 2
3. 3
4. > 3

Do you use guidelines to help develop the EOL simulation?

1. Yes
2. No <skip next question>

What guidelines are used? (Tick all that apply)

1. University developed
2. INACSL
3. Other <comment box provided>

Do you use a standardised template to develop the EOL simulations?

1. Yes
2. No

Is the EOL simulation peer reviewed prior to use?

1. Yes
2. No

Is the EOL simulation pilot tested prior to use?

1. Yes
2. No

**Simulation Event/Scenario**

Is an EOL simulation script provided to guide the person who assumes the patient’s role?

1. Yes
2. No

Is an EOL simulation script provided to guide confederates (secondary person who assumes a role to support simulation facilitation. E.G.: registered nurse, medical practitioner, allied health, pharmacy)?

1. Yes
2. No

Is an EOL simulation script provided to participants in the role of family members?

1. Yes
2. No

How are participants in the role of family members prepared for this role?

1. separate pre-brief
2. verbal orientation
3. no preparation
4. Other <comment box provided>

Is an EOL simulation instructor’s guide developed for facilitators?

1. Yes
2. No <skip next question>

What is included in the instructor’s guide? (tick all that apply)

1. Learning objectives
2. Scenario introduction
3. Scenario progression
4. Equipment list (consumables, medications, documentation)
5. Participant handover
6. Triggers
7. Pre-brief prompts
8. Debrief prompts
9. Other <comment box provided>

What are the learning objectives of the EOL simulation? (tick all that apply)

1. Communication to the patient
2. Communication to the family
3. Communication to inter-professional team
4. Pain management
5. Symptom management
6. Principles of loss, grief, and bereavement
7. Care during the silver hour (when death is recognised as an imminent possibility)
8. Family-oriented care
9. Strategies to support practitioner health and well being
10. Other <comment box provided>

Does the simulator die in the EOL simulation scenario?

1. Yes
2. No

How many students enter a segment/frame at once?

1. 1-2
2. 3-4
3. 5-6
4. > 6

What other roles are included in your EOL simulation? (tick all that apply)

1. Family member
2. Doctor
3. Social worker
4. Physiotherapist
5. Occupational therapist
6. Chaplain
7. Other <comment box provided>

Who plays the role of the family member?

1. Student
2. Facilitator
3. Actor
4. Other
5. NA

What roles do students play in the scenario? (tick all that apply)

1. Student nurse
2. Registered nurse
3. Family member
4. Doctor
5. Allied health
6. Patient
7. Other <comment box provided>

**Pre-brief**

Do you pre-brief students prior to the EOL simulation?

1. Yes
2. No

What is the duration of the pre-brief?

1. 0-10 minutes
2. 11 - 20 minutes
3. 21 - 30 minutes
4. > 30 minutes

What is included in your pre-brief? (tick all that apply)

1. Simulator orientation
2. Environment orientation
3. Scenario ISBAR handover
4. Learning objectives
5. Student expectations (confidentiality, code of conduct, participation, respect)
6. Student roles
7. Facilitator roles
8. Other <comment box provided>

Are students informed the simulator will die?

1. Yes
2. No
3. NA

**Debrief**

Do you debrief students after the EOL simulation?

1. Yes
2. No

What is the duration of the debrief?

1. 0-10 minutes
2. 11-20 minutes
3. 21-30 minutes
4. > 30 minutes

Which location does your debriefing take place?

1. Pre-brief room
2. Observation room
3. Lecture room
4. Lab room
5. Simulation scenario room

Which debrief model is used? (tick all that applies)

1. Plus-Delta Model
2. PEARLS reflection Model
3. Advocacy/Inquiry Model
4. Video Replay
5. Structured interview guide
6. Debriefing for meaningful learning Model
7. Other <comment box provided>

At which point of the simulation does debriefing occur?

1. After each segment/frame of the scenario
2. After the entire simulation scenario
3. Both

Is a script provided to assist with debrief?

1. Yes
2. No

Are counselling services offered to students?

1. Yes
2. No

**Facilitator Preparation**

Who facilitates into the EOL simulation? (tick all that applies)

1. Sessional academic
2. Lecturer in Nursing
3. Clinical educators
4. Medical staff
5. Allied Health staff
6. Other <comment box provided>

What standard preparation from the institution are facilitators provided with or expected to complete prior to facilitating simulations in general? (tick all that apply)

1. Simulation study day
2. Online simulation module
3. External training program e.g.: NHET-Sim
4. None
5. Other <comment box provided>

What content preparation from the institution are facilitators provided with prior or expected to complete prior to the EOL simulation? (tick all that applies)

1. Online training modules
2. University study day
3. Simulation instructor&#39;s guide
4. None
5. Other <comment box provided>

Are facilitators required to have any post graduate qualification in palliative care?

1. Yes
2. No

Are facilitators required to have clinical expertise in palliative care nursing?

1. Yes
2. No

<End of Survey>
